# Novel Systemic Inflammatory Markers Predict All-Cause Mortality in Patients Undergoing Endovascular Abdominal Aortic Aneurysm Repair

**DOI:** 10.31083/j.rcm2506202

**Published:** 2024-05-31

**Authors:** Wen-Xin Zhao, Zhi-Yuan Wu, Ning Zhao, Yong-Peng Diao, Yong Lan, Yong-Jun Li

**Affiliations:** ^1^Department of Vascular Surgery, Beijing Hospital, National Center of Gerontology, Institute of Geriatric Medicine, Chinese Academy of Medical Sciences, 100010 Beijing, China; ^2^Graduate School of Peking Union Medical College, Chinese Academy of Medical Sciences, 100010 Beijing, China

**Keywords:** novel systemic inflammatory markers, hemoglobin-to-red-cell distribution width ratio, abdominal aortic aneurysm, all-cause mortality

## Abstract

**Background:**

Clinically useful predictors for risk stratification of long-term survival may assist in selecting patients for endovascular abdominal aortic aneurysm (EVAR) procedures. This study aimed to analyze the prognostic significance of peroperative novel systemic inflammatory markers (SIMs), including the neutrophil-to-lymphocyte ratio (NLR), platelet-to-lymphocyte ratio (PLR), hemoglobin-to-red cell distribution width ratio (HRR), systemic immune-inflammatory index (SIII), and systemic inflammatory response index (SIRI), for long-term mortality in EVAR.

**Methods:**

A retrospective analysis was performed on 147 consecutive patients who underwent their first EVAR procedure at the Department of Vascular Surgery, Beijing Hospital. The patients were divided into the mortality group (n = 37) and the survival group (n = 110). The receiver operating characteristic curves were used to ascertain the threshold value demonstrating the most robust connection with mortality. The Kaplan–Meier survival analysis was performed between each SIM and mortality. The relationship between SIMs and survival was investigated using restricted cubic splines and multivariate Cox regression analysis.

**Results:**

The study included 147 patients, with an average follow-up duration of 34.28 ± 22.95 months. Deceased patients showed significantly higher NLR (*p* < 0.001) and reduced HRR (*p* < 0.001). The Kaplan–Meier estimates of mortality were considerably greater in the higher-NLR group (NLR >2.77) and lower-HRR group (HRR <10.64). The hazard ratio (HR) of 0.833 (95% confidence interval (95% CI): 0.71–0.97, *p* < 0.021) was determined to be statistically significant in predicting death in the multivariable analysis.

**Conclusions:**

Preoperative higher-NLR and lower-HRR have been associated with a lower long-term survival rate in abdominal aortic aneurysm (AAA) patients undergoing elective EVAR. Multivariate Cox regression showed that decreased preoperative HRR is an independent risk factor that increases mortality risk following EVAR. SIMs, such as the NLR and HRR, could be used in future clinical risk prediction methodologies for AAA patients undergoing EVAR. However, additional prospective cohort studies are needed to identify these findings.

## 1. Introduction

An abdominal aortic aneurysm (AAA) is characterized by an irreversible and progressive dilation of the abdominal aorta and often presents an 80% mortality rate after rupturing occurs [1, 2, 3]. Currently, the main surgical methods for treating an AAA are open surgical repair (OSR) and endovascular aortic repair (EVAR) [4]. Strong evidence from large randomized controlled trials has confirmed that EVAR is associated with reduced short-term mortality rates similar to mid- and long-term survival rates, although with higher reintervention rates during follow-up [5, 6, 7, 8]. Choosing a suitable surgical strategy is important to evaluate carefully the additional risk factors influencing prognosis, the patient’s anatomical characteristics, and preferences regarding follow-up [9, 10]. In this context, it is necessary to discover new, easily accessible, and generally applicable indicators for risk stratification in long-term survival conditions, thus, guiding treatment decisions [11].

Systemic chronic inflammation status is evaluated using novel systemic inflammatory markers (SIMs) derived from the whole blood cell count ratio. These markers include the neutrophil-to-lymphocyte ratio (NLR), platelet-to-lymphocyte ratio (PLR), hemoglobin-to-red cell distribution width ratio (HRR), systemic immune-inflammatory index (SIII), and systemic inflammatory response index (SIRI) [12, 13, 14]. These markers can more accurately indicate systemic inflammation for prognostic assessment [13]. Multiple studies have demonstrated a correlation between the novel SIMs and cardiovascular disease [15, 16], cancer prognosis [14, 17, 18, 19], and all-cause mortality [20]. The formation and development of the abdominal aorta are also closely related to the systemic inflammatory response [21, 22], and a cohort study has demonstrated a strong correlation between higher neutrophil count and abdominal aortic dissection [23]. Therefore, novel SIMs, which can serve as potential prognostic markers that are easy to obtain and widely used, are expected to be useful tools for identifying patients with poor survival outcomes after EVAR.

NLR has been proven to be associated with the perioperative morbidity of ruptured AAA and mortality after selective EVAR [24, 25]. However, the correlation between novel SIMs and the long-term survival rates after EVAR remains uncertain. Therefore, this study aimed to ascertain the accuracy of novel SIMs as prognostic indicators for predicting all-cause mortality in patients following EVAR.

## 2. Materials and Methods

### 2.1 Study Design, Inclusion, and Exclusion Criteria

A retrospective review of medical records showed 147 sequential individuals who underwent their first EVAR procedure at Beijing Hospital’s Vascular Surgery department between August 2016 and April 2023. The patients were mainly treated according to the criteria set by the European Society for Vascular Surgery (ESVS) [26, 27]. The surgeon made the final decision, considering the patient’s health characteristics and economic conditions.

The inclusion criteria were as follows: (1) patients diagnosed with AAA by computed tomography angiography (CTA), digital subtraction angiography (DSA), or ultrasound; (2) patients undergoing EVAR for the first time; (3) patients with complete perioperative and follow-up data; (4) patients who have provided informed consent and agreed to the intervention, including being told about other choices. The exclusion criteria include the following patients: (1) those who received conservative treatment, open surgical repair, or have presented as emergency cases; (2) those who have primary or secondary infectious AAA, abdominal aortic stent infection; (3) those who had symptomatic or ruptured AAA; (4) those who had congenital diseases: Marfan syndrome, *etc*.; (5) those who had an endoleak after endovascular treatment. This study was conducted with the approval of the Institutional Review Board.

### 2.2 Interventions and Baseline Characteristics 

After a detailed assessment and personalized treatment strategy, all patients underwent endovascular repair for AAA under either general or local anesthesia. Perioperative information, including demographic information, comorbidities (including the history of smoking, hypertension, diabetes, coronary heart disease, and related surgery, hyperlipidemia, chronic obstructive pulmonary disease, chronic renal insufficiency), a preoperative complete blood count, serum creatinine and serum albumin levels, maximum diameter, interventional data, postoperative complications, and follow-up information were collected and recorded.

### 2.3 Novel Systemic Inflammatory Markers

Novel SIMs, including NLR, PLR, SII, SIRI, and HRR, were calculated by preoperative whole blood count. The calculation formula for each is shown in Table 1. The receiver operating characteristic curve (ROC) was used to evaluate the diagnostic potential of each novel SIM, and the patients were divided into subgroups for subsequent analysis based on the cut-off value calculated by the Youden index.

**Table 1.** **Calculation formula for each index**.

Novel SIMs	Computing method
NLR	Neutrophil count (N)/lymphocyte count (L)
PLR	Platelet cell count (P)/lymphocyte count (L)
SII	Neutrophil count (N) × platelet count (P)/lymphocyte count (L)
SIRI	Neutrophil count (N) × monocyte count (M)/lymphocyte count (L)
HRR	Hemoglobin (Hb)/red cell distribution width (RDW)

SIMs, systemic inflammatory markers; NLR, neutrophil-to-lymphocyte ratio; PLR, platelet-to-lymphocyte ratio; SII, systemic immune-inflammation index; SIRI, systemic inflammation response index; HRR, hemoglobin-to-red cell distribution width.

### 2.4 Follow-up and Endpoints

After discharge, patients were regularly monitored by ultrasound, contrast-enhanced ultrasound, or CTA. The survival status was mainly obtained by telephone or outpatient follow-up. Death was considered as the endpoint event, and patients were monitored until either the event occurred or censorship took place. Patients who did not have an endpoint event were considered until their last follow-up (April 2023), and the average duration of each follow-up was calculated.

### 2.5 Statistical Analysis

Categorical variables are shown as counts and percentages and were compared using either Chi-squared or Fisher’s exact tests, depending on the circumstances. Continuous variables were presented as mean ± standard deviation (SD) or median (interquartile range, IQR) depending on their distribution and were compared using either the *t*-test or the Wilcoxon rank-sum test. Restricted cubic spline models with 3 knots were used to explore the relationship between novel SIM values and the survival after EVAR in Cox proportional hazards models. This method is a flexible statistical strategy that uses the observed data to discover the most suitable mathematical relationship between exposure and response. It provides a *p*-value to assess whether the relationship is linear or nonlinear. The predictive ability was assessed by calculating the area under the receiver operating characteristic curve (AUC). The best cut-off values for novel SIMs related to survival were determined as those that maximized the Youden index. Patient categorization into lower- and higher-novel SIMs groups was performed using a cut-off value for comparison. Survival was assessed by Kaplan–Meier survival analysis, and differences were compared using the log-rank test. We also conducted both univariable and multivariable analyses using Cox regression, considering the period at risk and including variables that differed significantly in the univariate analysis and multivariable logistic regression models. A *p*-value < 0.05 on both sides was considered statistically significant. Statistical analyses were performed using IBM SPSS 25.0 (IBM Crop, Chicago, IL, USA), GraphPad Prism 9.5.0 (GraphPad Software, San Diego, CA, USA), and R version 4.3.1 (R Foundation, Vienna, Austria).

## 3. Results

### 3.1 Patient Characteristics and Preoperative Complete Blood Results

The study included a cohort of 147 patients who underwent elective EVAR for AAA. Table 2 displays the clinical demographics and baseline characteristics of the participants in this investigation. The average age was 72.24 ± 8.59 years old, and most were male (83.07%). Among the participants, 44.26% were ever (current and past) smokers. The most prevalent comorbidities in this cohort were hypertension (72.79%, 107/147), coronary artery disease (47.62%, 70/147), and hyperlipidemia (29.25%, 43/147). The median AAA diameter was 55.0 mm (50.0–65.0). Table 2 displays the results of the preoperative whole blood test, which revealed no significant abnormalities.

**Table 2.** **Perioperative complications and deaths for the entire patient cohort following endovascular AAA repair**.

Variable			Overall (N = 147)
30-day mortality			4 (2.70%)
	Coronary artery syndrome		3 (2.04%)
	Gastrointestinal bleeding		1 (0.68%)
30-day complications			17 (11.5%)
	Hemorrhage		4 (2.70%)
		Gastrointestinal hemorrhage	2 (1.35%)
		Puncture site hematoma	2 (1.35%)
	Pseudoaneurysm		1 (0.68%)
	Radiographic contrast nephropathy		2 (1.35%)
	Myocardial infarction		5 (3.38%)
	Congestive heart failure		4 (2.70%)
	Respiratory failure		2 (1.35%)
	Gastrointestinal ischemia		4 (2.70%)

AAA, abdominal aortic aneurysm.

### 3.2 Perioperative Complications and Death

The incidence of perioperative complications was 11.5% (17/147). Four patients died during the perioperative period; three of them died of acute coronary syndrome, and one died of upper gastrointestinal bleeding due to postoperative stress, as shown in Table 2.

### 3.3 Differences between the Mortality Group and the Survival Group

The median duration of follow-up was 34.28 ± 22.95 months, ranging from 0 to 84 months. Participants were divided into survival and mortality groups based on outcome events. During the follow-up period, 37 patients (37/147, 25.17%) died, of which 15 patients (15/37, 40.54%) died due to cardiovascular and cerebrovascular events. The demographics and baseline characteristics of the two groups are outlined in Table 2. The mortality group showed a higher average age (*p* < 0.001) and a lower body mass index (BMI) (*p* = 0.007). More patients in the mortality group had chronic obstructive pulmonary disease (COPD) (*p* = 0.020), chronic renal insufficiency (*p* = 0.015), and higher preoperative creatinine (*p* = 0.001). The two groups had no significant difference in the incidence of other complications.

**Fig. 1.**
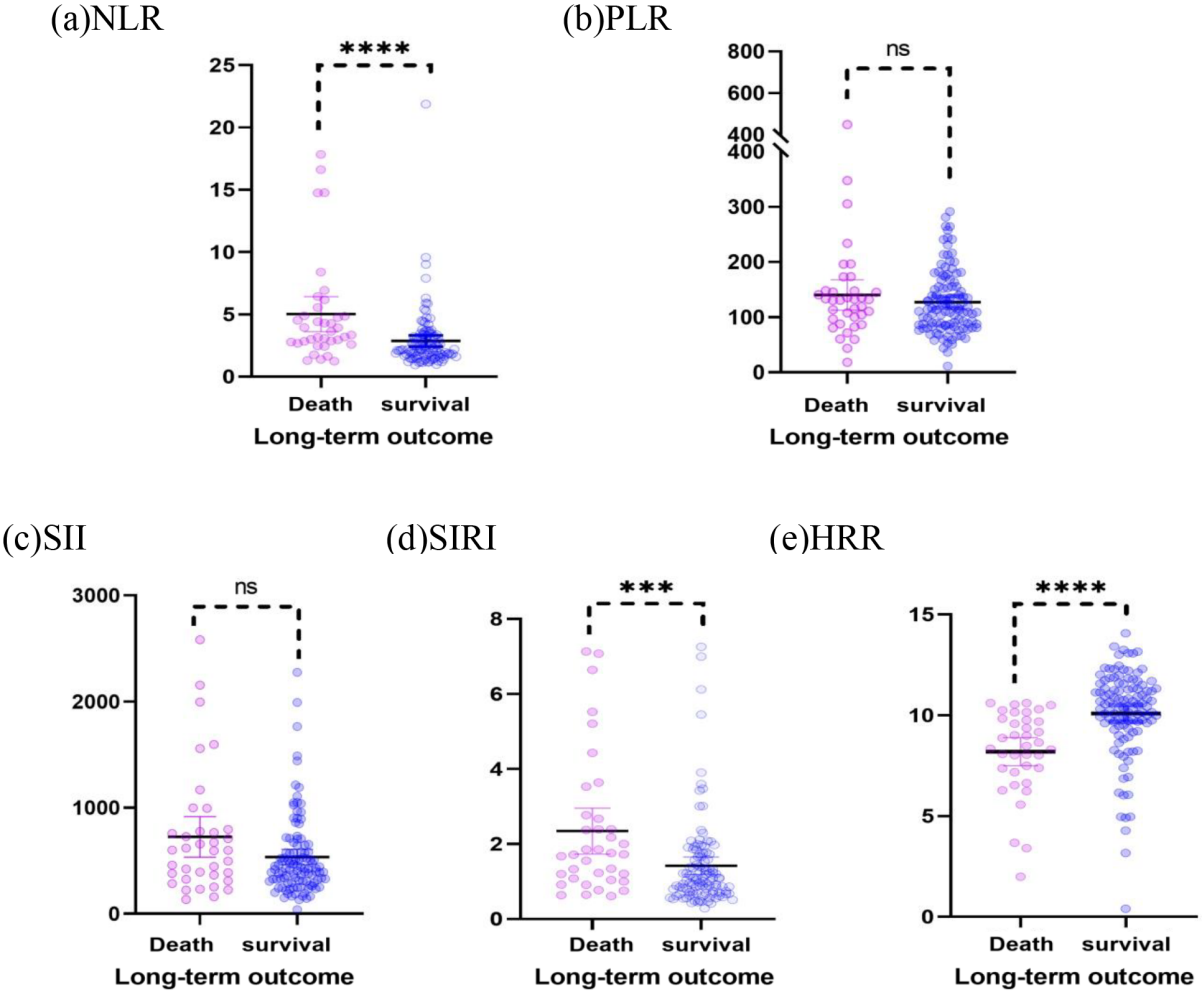
**Scatter plots of the NLR, PLR, SII, SIRI, and HRR show the distribution in the survival group (n = 110) and mortality group (n = 37).** (a) The NLR of the death group was higher than that of the survival group (*p* < 0.000). (b) The PLR of the mortality group was similar to that of the survival group (*p* = 0.444). (c) The SII of the mortality group was similar to that of the survival group (*p* = 0.072). (d) The SIRI of the mortality group was higher than that of the survival group (*p* = 0.001). (e) The HRR of the mortality group was higher than that of the survival group (*p* < 0.000). NLR, neutrophil-to-lymphocyte ratio; PLR, platelet-to-lymphocyte ratio; SII, systemic immune-inflammation index; SIRI, systemic inflammation response index; HRR, hemoglobin-to-red cell distribution width. *** indicates the *p*-value is less than 0.001; **** indicates the *p*-value is less than 0.000; ns indicates not statistically significant.

The results of the preoperative whole blood examination indicated that the mortality group had significantly lower preoperative hemoglobin levels (*p* < 0.001), lower preoperative albumin levels (*p* < 0.001), and higher platelet counts (*p* = 0.003). The mortality group had significantly higher NLR (*p* < 0.001) and SIRI (*p* = 0.001) but lower HRR (*p* < 0.001) compared to the survival group, as shown in Table 3 and Fig. 1.

**Table 3.** **Demographics and clinical characteristics for the entire patient cohort following endovascular AAA repair**.

Variable	All (N = 147)	Survival (N = 110)	Death (N = 37)	*p*-value
Demographic characteristics				
Age, years	72.24 ± 8.59	70.52 ± 7.80	77.38 ± 8.90	0.000*
Sex, male	128 (83.07%)	97 (88.18%)	31 (83.78%)	0.490
BMI, kg/$m^{2}$	24.34 ± 3.84	24.83 ± 3.81	22.87 ± 3.58	0.007*
Medical history and comorbidities				
Smoking history	67 (44.26%)	50 (45.45%)	17 (45.95%)	0.959
Hypertension	107 (72.79%)	78 (70.91%)	29 (78.39%)	0.377
Diabetes	26 (17.69%)	18 (16.36%)	8 (21.62%)	0.468
Hyperlipidemia	43 (29.25%)	32 (29.09%)	11 (29.73%)	0.941
COPD	11 (7.48%)	5 (4.55%)	6 (16.21)	0.020*
Coronary artery disease	70 (47.62%)	50 (45.45%)	20 (54.05%)	0.365
Myocardial infarction	33 (22.45%)	26 (23.64%)	7 (18.92%)	0.552
Prior CABG	7 (4.76%)	6 (5.45%)	1 (2.70)	0.497
Prior PCI	25 (17.01%)	21 (19.09%)	4 (10.81%)	0.246
Cerebrovascular accident	25 (17.01%)	16 (14.55%)	9 (24.32)	0.171
Chronic renal impairment	16 (10.88%)	8 (7.27%)	8 (21.62)	0.015*
Clinical features				
WBCs (×${\mathrm{10}}^{3}$/mL)	6.18 (5.35–7.74)	6.09 (5.26–7.55)	6.56 (5.66–8.06)	0.252
Neutrophil count (×${\mathrm{10}}^{3}$/mL)	3.84 (3.11–5.03)	3.69 (3.08–4.55)	4.78 (3.49–6.15)	0.006*
Lymphocyte count (×${\mathrm{10}}^{3}$/mL)	1.50 (1.14–2.02)	1.66 (1.19–2.13)	1.22 (1.03–1.62)	0.001*
Monocyte count (×${\mathrm{10}}^{3}$/mL)	0.49 (0.39–0.57)	0.49 (0.38–0.57)	0.48 (0.40–0.56)	0.975
Red cell distribution width	12.9 (12.5–13.5)	12.8 (12.4–13.4)	13.2 (12.65–14.45)	0.054
Hemoglobin (mg/dL)	129.0 (115.25–141.0)	136.0 (120.0–144.0)	116.0 (98.50–124.50)	0.000*
Platelets (n/µL)	181.0 (146.0–220.0)	186.0 (158.0–231.5)	161.0 (106.5–191.5)	0.003*
NLR	2.58 (1.79–3.70)	2.18 (1.70–3.31)	3.63 (2.72–5.21)	0.000*
PLR	116.07 (85.95–155.45)	113.84 (84.54–157.52)	130.68 (92.33–147.8)	0.444
SII	445.33 (311.03–708.63)	426.21 (305.83–646.41)	597.73 (345.26–786.11)	0.072
SIRI	1.20 (0.77–1.94)	1.06 (0.70–1.74)	1.72 (1.06–2.72)	0.001
HRR	10.08 (8.35–11.20)	10.48 (9.44–11.52)	8.49 (7.28–9.80)	0.000*
Creatinine (mmol/L)	83.0 (77.50–104.0)	80.0 (70.0–99.0)	99.5 (78.0–130.5)	0.001
Albumin (g/L)	39.0 (36.0–41.0)	39.0 (37.0–41.0)	36.0 (34.5–38.5)	0.000*
Aneurysm diameter (mm)	55.0 (50.0–65.0)	55.0 (48.0–60.0)	64.0 (54.0–75.0)	0.001*

* indicates the *p*-value is less than 0.05. Data are presented as n (%), mean ± standard deviation, or median (interquartile range, IQR). BMI, body mass index; COPD, chronic obstructive pulmonary disease; WBCs, white blood cells; CABG, coronary artery bypass grafting; PCI, percutaneous coronary intervention.

### 3.4 ROC Analysis and Kaplan–Meier Survival Curves Evaluating the Novel SIMs to Predict Death after Elective EVAR

**Fig. 2.**
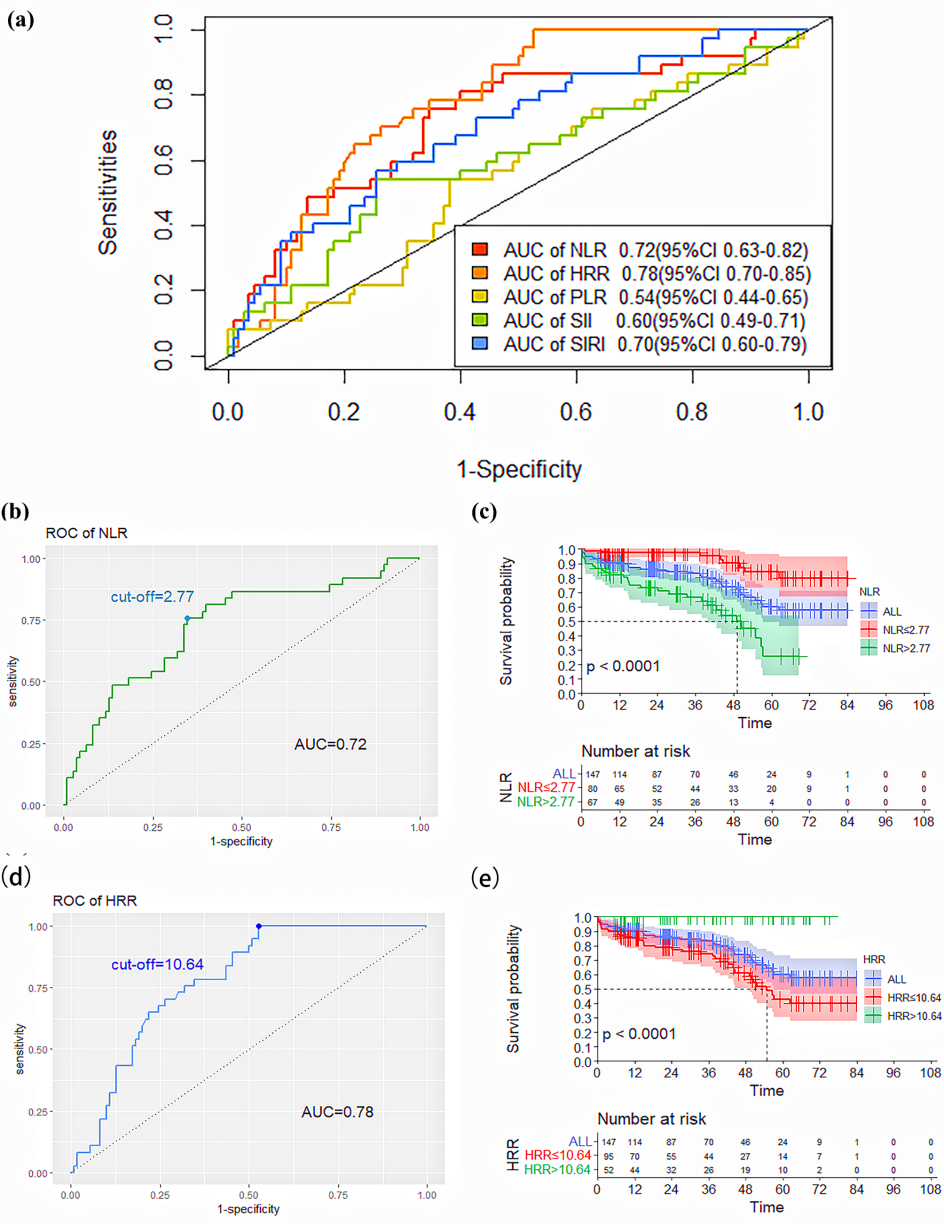
**ROC curves of the NLR, PLR, SII, SIRI, and HRR for predicting death.** (a) ROC curves of the NLR, PLR, SII, SIRI, and HRR for predicting mortality. (b) ROC curves and the cut-off value of the NLR for predicting mortality. (c) Kaplan–Meier survival curves of the lower-NLR and higher-NLR groups (*p* < 0.0001). (d) ROC curves and the cut-off value of the HRR for predicting mortality. (e) Kaplan–Meier survival curves of the lower-NLR and higher-NLR groups (*p* < 0.0001). ROC, receiver operating characteristic curve; AUC, the area under the receiver operating characteristic curve.

The ROC curve was used to analyze the predictive ability of novel SIMs for death after elective EVAR. Based on the AUC value, we found that NLR (AUC: 0.72, 95% confidence interval (95% CI): 0.63–0.82) and HRR (AUC: 0.78, 95% CI: 0.70–0.85) had significantly higher abilities to predict patient death compared to PLR (AUC: 0.54, 95% CI: 0.44–0.65), SII (AUC: 0.60, 95% CI: 0.49–0.71) and SIRI (AUC: 0.70, 95% CI: 0.60–0.79), as shown in Table 4 and Fig. 2a. The NLR and HRR values corresponding to the greatest value of the Youden index were calculated as the ideal cut-off points on the ROC curve of death.

**Table 4.** **Receiver operating characteristic curve analysis**.

Variable	AUC (95% CI)	Cut-off point	Sensitivity	Specificity
NLR	0.72 (0.63–0.82)	2.77	0.76	0.65
HRR	0.78 (0.70–0.85)	10.64	1.00	0.47
PLR	0.54 (0.44–0.65)	130.30	0.54	0.62
SII	0.60 (0.49–0.71)	589.12	0.54	0.75
SIRI	0.70 (0.60–0.79)	1.67	0.58	0.75

95% CI, 95% confidence interval; AUC, the area under the receiver operating characteristic curve.

The ROC curve analysis showed that an NLR value of 2.77 was the calculated cut-off point for predicting death, with a sensitivity of 76% and a specificity of 65%. According to the cut-off value, patients were divided into the lower-NLR (<2.77) and the higher-NLR (>2.77) groups. Of the 37 patients who died, 28 (75.68%) belonged to the higher-NLR group, as shown in Table 3 and Fig. 2b. The Kaplan–Meier survival curve revealed a significant association between the higher-NLR group and an elevated risk of death (*p* < 0.001), as shown in Fig. 2c.

Moreover, the determined threshold for HRR was 10.64. Further, HRR <10.64 was strongly linked to a higher risk of death, as shown in Table 3 and Fig. 2d,e. The patients were classified based on ideal cut-off values for SIMs, and a subgroup analysis was conducted to examine the cause-specific and all-cause deaths of the patients. We found a significant increase in all-cause and cardiovascular mortality in the higher-NLR and lower-HRR groups (*p* < 0.05). However, there was no significant variation in mortality for any other causes, as indicated in Table 5.

**Table 5.** **Comparison of all-cause death between the lower-/higher-NLR and HRR groups**.

Variable		Overall (N = 147)	NLR <2.77 (N = 81)	NLR ≥2.77 (N = 66)	*p*-value
All cause death		37 (25.17%)	9 (11.11%)	28 (42.42%)	0.000*
	Aneurysm-related death	2 (1.36%)	0 (0%)	2 (3.03%)	0.115
	MACE	15 (10.2%)	3 (3.7%)	12 (18.18%)	0.004*
	Renal failure	2 (1.36%)	1 (1.23%)	1 (1.52%)	0.884
	Multiple organ failure	5 (3.4%)	1 (1.23%)	4 (6.06%)	0.108
	Cancer	3 (2.04%)	1 (1.23%)	2 (3.03%)	0.444
	COVID-19	6 (4.08%)	2 (2.47%)	4 (6.06%)	0.247
	Others	4 (2.72%)	1 (1.23%)	3 (4.55%)	0.220
Variable		Overall (N = 147)	HRR <10.64 (N = 88)	HRR ≥10.64 (N = 59)	*p*-value
All cause death		37 (25.17%)	33 (37.5%)	4 (6.78%)	0.000*
	Aneurysm-related death	2 (1.36%)	2 (2.27%)	0 (0%)	0.224
	MACE	15 (10.2%)	15 (17.05%)	0 (0%)	0.001*
	Renal failure	2 (1.36%)	2 (2.27%)	0 (0%)	0.224
	Multiple organ failure	5 (3.4%)	5 (5.68%)	0 (0%)	0.062
	Cancer	3 (2.04%)	2 (2.27%)	1 (1.69%)	0.808
	COVID-19	6 (4.08%)	3 (3.41%)	3 (5.08%)	0.615
	Others	4 (2.72%)	4 (4.55%)	0 (0%)	0.097

* indicates the *p*-value is less than 0.05. Data are presented as n (%). MACE, major adverse cardiovascular event; COVID-19, coronavirus disease 2019.

**Fig. 3.**
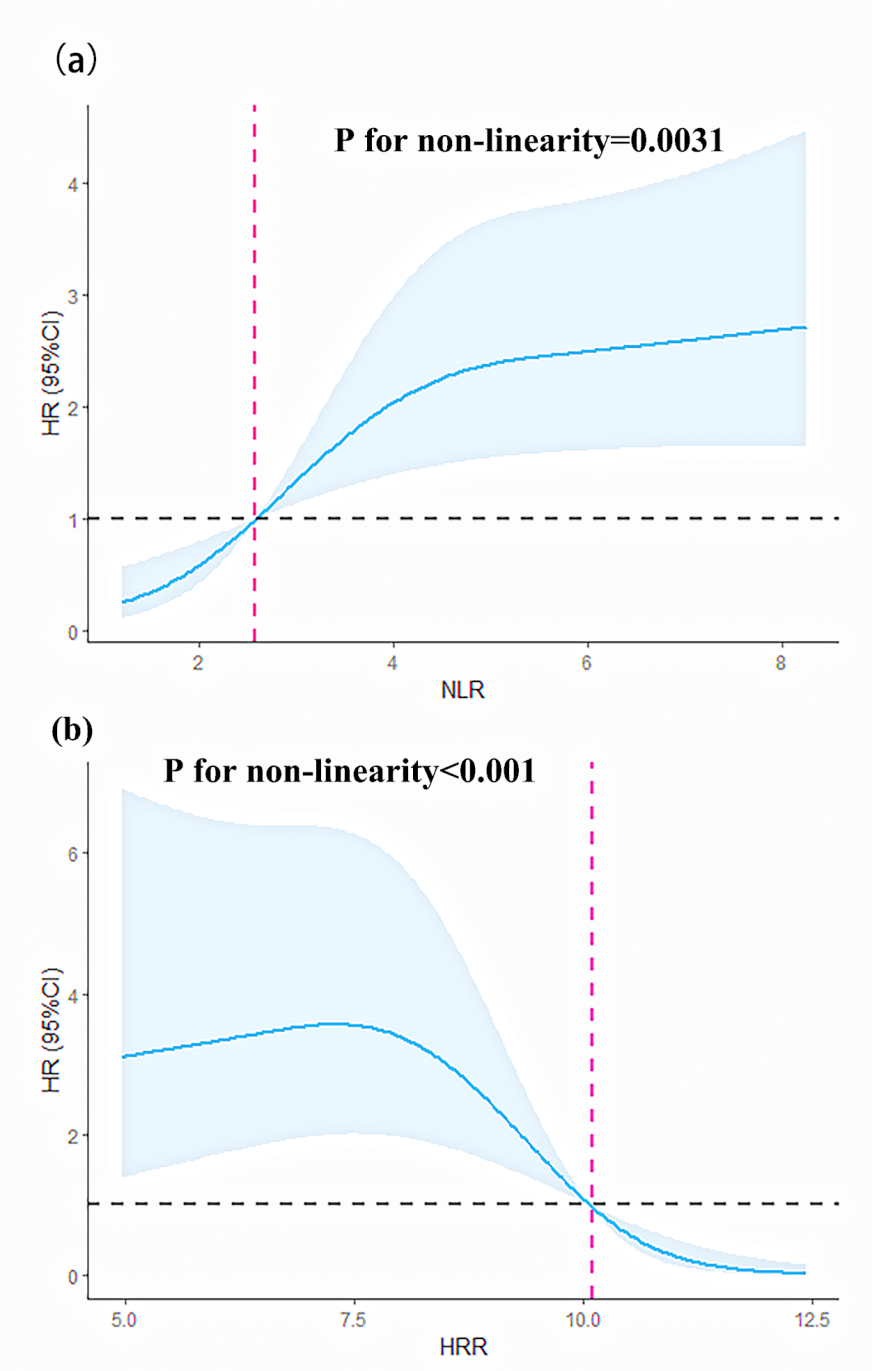
**Restricted cubic splines of the NLR and HRR for predicting HR.** (a) Restricted cubic splines of the nonlinear relationship between NLR and HR. (b) Restricted cubic splines of the nonlinear relationship between HRR and HR. 95% CI, 95% confidence interval; HR, hazard ratio.

We modeled and visualized the relationships of NLR/HRR and all-cause mortality using restricted cubic splines. The results showed that when the NLR >2.6, there was an elevated risk of mortality (*p*-value for non-linearity trend test = 0.0031). This was supported by the observation that the NLR value was 2.6, corresponding to a hazard ratio (HR) of 1. Similarly, when HR was equal to 1, it corresponded to an HRR of 10.10. Further, an HRR value of less than 10.10 was found to considerably increase the risk of all-cause mortality (*p*-value for non-linearity trend test < 0.0001), as shown in Fig. 3.

### 3.5 Univariate and Multivariate Cox Regression Analyses of the HR for Death after Elective EVAR

**Fig. 4.**
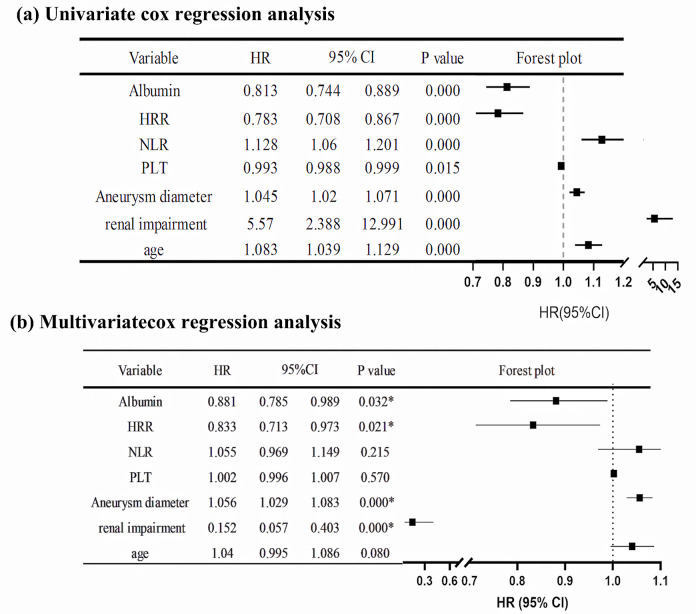
**Cox regression analysis of the hazard ratio for death after EVAR in AAA patients.** (a) Univariate Cox regression analysis of HR after EVAR in AAA patients. (b) Multivariate Cox regression analysis of HR after EVAR in AAA patients. * indicates the *p*-value is less than 0.05. 95% CI, 95% confidence interval; HR, hazard ratio; AAA, abdominal aortic aneurysm; PLT, platelets.

The variables included in the Cox regression analysis were age, gender, smoking history, hypertension, diabetes, hyperlipidemia, COPD, coronary heart disease, myocardial infarction, previous coronary artery bypass grafting (CABG), previous percutaneous coronary intervention (PCI), cerebrovascular accident, chronic renal insufficiency, hemoglobin, platelet count, NLR, HRR, albumin, and aneurysm diameter. The results of the univariate Cox regression analysis showed that age (HR = 1.083, *p* < 0.001), renal impairment (HR = 5.57, *p* < 0.001), platelets (PLT) (HR = 0.993, *p* < 0.015), NLR (HR = 1.128, *p* < 0.001), HRR (HR = 0.783, *p* < 0.001), albumin (HR = 0.813, *p* < 0.001), and aneurysm diameter (HR = 1.045, *p* < 0.001) were risk factors for death. After eliminating collinearity and considering clinical experience, factors that demonstrated a *p*-value < 0.05 in the univariate Cox regression analysis were included in the multivariate Cox regression analysis. Results showed that renal impairment (HR = 0.152, *p* < 0.001), HRR (HR = 0.833, *p* = 0.021), albumin (HR = 0.881, *p* = 0.032), and aneurysm diameter (HR = 1.056, *p* < 0.001) were the independent risk factors for death, as shown in Fig. 4.

## 4. Discussion

We presented a detailed analysis of clinical factors and hematologic markers that have predictive significance for death in AAA patients undergoing EVAR, particularly the relationship between SIMs and long-term death. The results suggested that an increased preoperative NLR and a decreased HRR were associated with increased death after EVAR. AAA patients with an NLR ≥2.77 or HRR <10.64 may have a higher risk of death during the follow-up after EVAR. In addition, multivariate Cox regression analysis suggested that HRR is an independent risk factor for death after EVAR, with a prediction accuracy of 0.78.

Recently, SIMs have received considerable attention as independent prognostic indicators for mortality and morbidity in several diseases, such as cancers, cardiovascular and cerebrovascular diseases, and inflammatory conditions [28, 29, 30, 31]. Thus, using SIMs as inexpensive and easily accessible prognostic indicators for follow-up is steadily increasing in clinical and academic settings. The application of NLR in the prognosis of mortality and morbidity in coronary artery disease [32], atherosclerosis [15], and peripheral artery disease [33] has been widely studied. In terms of coronary artery disease, higher NLR values can predict not only the progression of coronary atherosclerosis [34] but also the risk of death after CABG and PCI [35, 36]. In addition, in five randomized trials involving 600,875 participants, NLR has been a proven predictor of incident major adverse cardiovascular events (MACEs) and all-cause mortality [15]. However, SII and SIRI use three blood cell subtypes and might provide a more accurate representation of the balance between inflammatory and immunological responses. Several studies have shown that elevated SII is associated with an increased incidence and severity of coronary heart disease [37, 38] and with a higher risk of MACEs (HR: 1.65) and total major events (HR: 1.53) in coronary artery disease (CAD) patients [39]. Similarly, SIRI was an independent predictor of MACEs in acute coronary syndrome (ACS) patients undergoing PCI [40]. However, there are relatively limited studies on HRR. In a retrospective study of 6046 hospitalized coronary atherosclerotic heart disease patients undergoing PCI, decreased levels of HRR (HRR <10.25) were associated with a 1.470-fold and 1.479-fold higher risk of long-term all-cause and cardiac mortality, respectively [41]. SIMs provide significant potential for application in cardiovascular disease prognosis research.

The predictive significance of SIMs in aortic-related surgery has also received increasing attention. Several studies have proposed the predictive value of SIMs for long-term survival after EVAR. King *et al*. [24] found that preoperative NLR (NLR >4) was a strong independent predictor of mid-term mortality after EVAR. Kordzadeh *et al*. [42] showed that a preoperative NLR >5, irrespective of age, gender, AAA size, blood loss, length of stay, and comorbidities is an independent marker of 30-day death in patients with a ruptured AAA. The study by Lecumberri *et al*. [25] revealed that NLR, PLR, and SII exhibited independent associations with survival outcomes in a 5-year follow-up of AAA patients who underwent EVAR. However, only NLR demonstrated a moderate enhancement in predicting a survival score. Hence, an NLR ≥3 can be used to identify patients with poor outcomes and help in decision-making [25]. In addition, Zhao *et al*. [12] conducted a survival study and found that patients in the higher-SIRI group (SIRI >4) had a 4.3 times higher risk of experiencing adverse outcomes after EVAR compared to patients with a lower-SIRI. However, it is important to note that the optimal thresholds selected by different research teams for various characteristics in the prognosis of SIMs in AAA exhibit significant heterogeneity. Currently, there is no widely agreed upon threshold of SIMs to predict perioperative complications, mid- and long-term survival, and length of hospital stay of AAA patients following EVAR. Therefore, further supplementation and improvement of relevant studies are required. We comprehensively investigated the prognostic value of SIMs, while to the best of our knowledge, this is the first study that assessed the relationship between HRR and long-term survival after EVAR.

NLR represents a chronic, mild systemic inflammatory response, often accompanied by elevated levels of proinflammatory cytokines. This reaction enables the body to respond to inflammatory stimuli, activating inflammatory cells within the plaque and leading to a catastrophic cascade. Conversely, chronic inflammation appears to be significantly involved in the development of AAA [43]. Inflammatory cells, such as neutrophils, can generate oxygen-derived free radicals that can trigger apoptosis and induce phenotypic alterations in vascular smooth muscle cells. This process eventually results in a partial decline in the production and repair capability of the vascular matrix [44]. In addition, proteases secreted by inflammatory cells such as neutrophils might result in the fragmentation of microfibrils inside the matrix, ultimately causing a reduction in the elasticity of the cell wall [45]. When the extracellular matrix (ECM) structure is destroyed and the media loses its elasticity, soluble blood components, including various inflammatory cells, can move and build up in the media through the highly vascularized adventitia. This, combined with platelet aggregation and coagulation system activation, encourages the development of luminal thrombosis. As a result, the aorta dilates and becomes more susceptible to rupturing in cases of AAA [46]. On the other hand, the development of atherothrombosis relies on the systemic chronic inflammatory response. The high neutrophil count is positively associated with the risk of plaque rupturing [47, 48] and increases the risk of microcirculation thrombosis [49]. Monocytes also play a role in initiating and promoting atherosclerosis, and their counts have been described as predictors of cardiovascular diseases (CVD) mortality, independent of other classical risk factors [50, 51]. Lymphopenia is an immunosuppressive and adverse physiologically stressful state that is associated with poor outcomes [52, 53]. Therefore, a rise in NLR may be linked to a high incidence of cardiovascular and cerebrovascular events, as supported by our subgroup analysis findings, which indicate that patients with a high NLR value had more cardiovascular and cerebrovascular deaths. Moreover, while NLR does not independently predict the postoperative prognosis of AAA, its predictive performance is relatively better than that of novel SIMs, such as PLR, SII, and SIRI. This may be attributed to the stability of NLR levels over time, as reported by Wang *et al*. [54]. In five contemporary randomized trials, Adamstein *et al*. [15] found that NLR levels remained stable over time among patients assigned to the placebo, and this consistency over time provides a clinical rationale for their use as a simple and reliable measure for follow-up. The study has also shown that medication interventions such as aspirin and statins may regulate NLR by reducing the inflammatory response through pleiotropic effects [15]. Patients with high preoperative NLR should be closely monitored for the development of cardiovascular and cerebrovascular events during the perioperative period and in the long term. Additionally, assessing the need for prolonged administration of lipid-lowering and antiplatelet medications for preventive purposes is important.

Lower hemoglobin is a crucial marker of potential inflammatory states and is associated with poor prognosis in several diseases [55]. In studies of AAAs, lower hemoglobin concentration is independently associated with higher probabilities of 30-day death, more in-hospital adverse outcomes, and reduced long-term survival after EVAR [56, 57]. Meanwhile, red cell distribution width (RDW) can reflect the underlying inflammatory state and is associated with adverse cardiovascular disease outcomes [58, 59]. Förhécz *et al*. [60] conducted a retrospective cohort study involving 195 patients diagnosed with chronic heart failure. The findings revealed a significant correlation between RDW and inflammatory markers, including C-reactive protein and other soluble cytokines [60]. A higher preoperative RDW level is linearly and high-risk associated with 5-year survival after EVAR [23]. The reason may be that inflammatory factors promote the formation of lysophosphatidylcholine in systemic inflammation, and increased phosphatidylserine exposure leads to lipid remodeling of the erythrocyte membrane, thereby impacting the function and longevity of erythrocytes. Inflammation accelerates the clearance of red blood cells (RBCs) by activating macrophages, reducing the life span of RBCs, and decreasing hemoglobin levels. An increase in RDW may reflect an elevation in the number of nonfunctional RBCs or the destruction of healthy cells [61, 62, 63]. The decrease in hemoglobin (Hb) represents the impaired oxygen-carrying function, while the rise in RDW reflects the negative effect of inflammation and other causes on the erythroid function of the bone marrow [41]. The HRR, calculated by Hb/RDW, reflects the superposition of these phenomena and has a broader range of applications. A meta-analysis reported the advantage of combining RDW with Hb for cardiovascular disease prognostic ratios, indicating that HRR is a highly effective strategy for predicting cardiovascular disease outcomes [64]. In addition, Qu *et al*. [65] analyzed 233 elderly patients with coronary heart disease (CHD) and found that HRR was a stronger predictor of frailty compared to hemoglobin or RDW; moreover, frailty was identified as a significant indication of prognostic factors for AAA. However, this measure has only been studied in a particular fraction of cancer and cardiovascular disease instances [66, 67, 68]. There is a lack of data on HRR in patients undergoing endovascular repair of AAAs. This is the first study to include HRR in the prognosis analysis of AAA, and it shows that decreased HRR indicates increased death after AAA surgery and can be used as an independent risk factor for long-term death. Subgroup analysis showed that cardiovascular and cerebrovascular mortality was more obviously increased in the lower-HRR group. Thus, the Hb/RDW ratio is a simple and practical prediction tool that can help clinicians estimate the risk stratification of EVAR patients.

Caution should still be exercised when considering using novel SIMs as a surgical consideration for AAA, as our studies have several limitations. First, as a single-center retrospective observational study, this study is limited by its relatively small sample size, leaving certain confounding factors unmeasured. Secondly, only preoperative whole blood cell counts were collected and used to calculate the SIMs, and there were no relevant data regarding the follow-up. Therefore, it is hard to study the impact of the variation in these SIMs, while their stability may also be uncertain. In previous studies, the cut-off values of inflammatory indicators in each system exhibited significant variation. There is a lack of consensus on the best threshold and the degree of association with various outcomes. Thirdly, the relevant pathophysiological mechanisms remain uncertain. Lastly, it should be emphasized that these SIMs have been reported to be associated with other types of cardiovascular diseases. Moreover, the use of SIMs as predictive markers for AAA patients has potential overlap and can make it be complicated by the fact that they can also be present in other types of aortic disease, such as thoracic aortic aneurysm, aortic dissection-renal aneurysm, and splenic aneurysm. These issues may hinder the clinical application of SIMs. Therefore, SIMs require additional comprehensive and carefully designed multicenter investigations with large sample sizes to validate these findings since they have the potential to serve as a valuable clinical tool for categorizing the risk of EVAR patients.

## 5. Conclusions

High preoperative NLR and low preoperative HRR indicate a decreased long-term survival rate of patients with an AAA after elective EVAR. HRR was identified as an independent risk factor for postoperative prognosis following elective EVAR via multivariate Cox regression. Patients whose HRR is below 10.64 should have perioperative and long-term cardiovascular and cerebrovascular events closely monitored, and the extension of anti-lipid and anti-platelet drug therapies should be considered necessary. However, further comprehensive and meticulously planned multicenter investigations are required to confirm these findings due to the existing limitations.

## Data Availability

The datasets used and/or analyzed during the current study are available from the corresponding author on reasonable request.

## References

[1] Golledge J, Muller J, Daugherty A, Norman P (2006). Abdominal aortic aneurysm: pathogenesis and implications for management. *Arteriosclerosis, Thrombosis, and Vascular Biology*.

[2] Golledge J (2019). Abdominal aortic aneurysm: update on pathogenesis and medical treatments. *Nature Reviews. Cardiology*.

[3] Reimerink JJ, van der Laan MJ, Koelemay MJ, Balm R, Legemate DA (2013). Systematic review and meta-analysis of population-based mortality from ruptured abdominal aortic aneurysm. *The British Journal of Surgery*.

[4] Chen ZG, Tan SP, Diao YP, Wu ZY, Miao YQ, Li YJ (2019). The long-term outcomes of open and endovascular repair for abdominal aortic aneurysm: A meta-analysis. *Asian Journal of Surgery*.

[5] Wu ZY, Chen ZG, Diao YP, Sun R, Liu CW, Chen YX (2019). Endovascular Repair of Complex Aortoiliac Aneurysm with the Sandwich Technique in Sixteen Patients. *Annals of Vascular Surgery*.

[6] De Bruin JL, Baas AF, Buth J, Prinssen M, Verhoeven ELG, Cuypers PWM (2010). Long-term outcome of open or endovascular repair of abdominal aortic aneurysm. *The New England Journal of Medicine*.

[7] Greenhalgh RM, Brown LC, Powell JT, Thompson SG, Epstein D, United Kingdom EVAR Trial Investigators (2010). Endovascular versus open repair of abdominal aortic aneurysm. *The New England Journal of Medicine*.

[8] Lederle FA, Freischlag JA, Kyriakides TC, Padberg FT, Matsumura JS, Kohler TR (2009). Outcomes following endovascular vs open repair of abdominal aortic aneurysm: a randomized trial. *JAMA*.

[9] Wu ZY, Chen ZG, Ma L, Diao YP, Chen YX, Liu CW (2017). Outcomes of Chimney and/or Periscope Techniques in the Endovascular Management of Complex Aortic Pathologies. *Chinese Medical Journal*.

[10] Hart T, Milner R (2016). Surgical Versus Endovascular Aortic Aneurysm Repair: Evidence to Guide the Optimal Approach for the Individual Patient. *Current Atherosclerosis Reports*.

[11] Khashram M, Williman JA, Hider PN, Jones GT, Roake JA (2016). Systematic Review and Meta-analysis of Factors Influencing Survival Following Abdominal Aortic Aneurysm Repair. *European Journal of Vascular and Endovascular Surgery: the Official Journal of the European Society for Vascular Surgery*.

[12] Zhao Y, Hong X, Xie X, Guo D, Chen B, Fu W (2022). Preoperative systemic inflammatory response index predicts long-term outcomes in type B aortic dissection after endovascular repair. *Frontiers in Immunology*.

[13] Labonté C, Zhand N, Park A, Harvey PD (2022). Complete blood count inflammatory markers in treatment-resistant schizophrenia: Evidence of association between treatment responsiveness and levels of inflammation. *Psychiatry Research*.

[14] Petrella F, Casiraghi M, Radice D, Cara A, Maffeis G, Prisciandaro E (2021). Prognostic Value of the Hemoglobin/Red Cell Distribution Width Ratio in Resected Lung Adenocarcinoma. *Cancers*.

[15] Adamstein NH, MacFadyen JG, Rose LM, Glynn RJ, Dey AK, Libby P (2021). The neutrophil-lymphocyte ratio and incident atherosclerotic events: analyses from five contemporary randomized trials. *European Heart Journal*.

[16] Fang Y, Sun X, Zhang L, Xu Y, Zhu W (2022). Hemoglobin/Red Blood Cell Distribution Width Ratio in Peripheral Blood Is Positively Associated with Prognosis of Patients with Primary Hepatocellular Carcinoma. *Medical Science Monitor: International Medical Journal of Experimental and Clinical Research*.

[17] Li B, Zhou P, Liu Y, Wei H, Yang X, Chen T (2018). Platelet-to-lymphocyte ratio in advanced Cancer: Review and meta-analysis. *Clinica Chimica Acta; International Journal of Clinical Chemistry*.

[18] Moon G, Noh H, Cho IJ, Lee JI, Han A (2020). Prediction of late recurrence in patients with breast cancer: elevated neutrophil to lymphocyte ratio (NLR) at 5 years after diagnosis and late recurrence. *Breast Cancer (Tokyo, Japan)*.

[19] Guo J, Fang J, Huang X, Liu Y, Yuan Y, Zhang X (2018). Prognostic role of neutrophil to lymphocyte ratio and platelet to lymphocyte ratio in prostate cancer: A meta-analysis of results from multivariate analysis. *International Journal of Surgery (London, England)*.

[20] Duffy BK, Gurm HS, Rajagopal V, Gupta R, Ellis SG, Bhatt DL (2006). Usefulness of an elevated neutrophil to lymphocyte ratio in predicting long-term mortality after percutaneous coronary intervention. *The American Journal of Cardiology*.

[21] Wu ZY, Trenner M, Boon RA, Spin JM, Maegdefessel L (2020). Long noncoding RNAs in key cellular processes involved in aortic aneurysms. *Atherosclerosis*.

[22] Hellenthal FAMVI, Geenen ILA, Teijink JAW, Heeneman S, Schurink GWH (2009). Histological features of human abdominal aortic aneurysm are not related to clinical characteristics. *Cardiovascular Pathology: the Official Journal of the Society for Cardiovascular Pathology*.

[23] Shah AD, Denaxas S, Nicholas O, Hingorani AD, Hemingway H (2017). Neutrophil Counts and Initial Presentation of 12 Cardiovascular Diseases: A CALIBER Cohort Study. *Journal of the American College of Cardiology*.

[24] King AH, Schmaier AH, Harth KC, Kumins NH, Wong VL, Zidar DA (2020). Elevated neutrophil-lymphocyte ratio predicts mortality following elective endovascular aneurysm repair. *Journal of Vascular Surgery*.

[25] Lecumberri E, Ruiz-Carmona C, Mateos E, Galarza A, Subirana I, Clara A (2021). Prognostic Value of Inflammatory Biomarkers in 5-Year Survival After Endovascular Repair of Abdominal Aortic Aneurysms in a Predominantly Male Cohort: Implications for Practice. *World Journal of Surgery*.

[26] Wanhainen A, Verzini F, Van Herzeele I, Allaire E, Bown M, Cohnert T (2019). Editor’s Choice - European Society for Vascular Surgery (ESVS) 2019 Clinical Practice Guidelines on the Management of Abdominal Aorto-iliac Artery Aneurysms. *European Journal of Vascular and Endovascular Surgery: the Official Journal of the European Society for Vascular Surgery*.

[27] Moll FL, Powell JT, Fraedrich G, Verzini F, Haulon S, Waltham M (2011). Management of abdominal aortic aneurysms clinical practice guidelines of the European society for vascular surgery. *European Journal of Vascular and Endovascular Surgery: the Official Journal of the European Society for Vascular Surgery*.

[28] Karimi A, Shobeiri P, Kulasinghe A, Rezaei N (2021). Novel Systemic Inflammation Markers to Predict COVID-19 Prognosis. *Frontiers in Immunology*.

[29] Savioli F, Morrow ES, Dolan RD, Romics L, Lannigan A, Edwards J (2022). Prognostic role of preoperative circulating systemic inflammatory response markers in primary breast cancer: meta-analysis. *The British Journal of Surgery*.

[30] Jin Z, Wu Q, Chen S, Gao J, Li X, Zhang X (2021). The Associations of Two Novel Inflammation Indexes, SII and SIRI with the Risks for Cardiovascular Diseases and All-Cause Mortality: A Ten-Year Follow-Up Study in 85,154 Individuals. *Journal of Inflammation Research*.

[31] Choi ES, Kim HS, Han I (2014). Elevated preoperative systemic inflammatory markers predict poor outcome in localized soft tissue sarcoma. *Annals of Surgical Oncology*.

[32] Dziedzic EA, Gąsior JS, Tuzimek A, Paleczny J, Junka A, Dąbrowski M (2022). Investigation of the Associations of Novel Inflammatory Biomarkers-Systemic Inflammatory Index (SII) and Systemic Inflammatory Response Index (SIRI)-With the Severity of Coronary Artery Disease and Acute Coronary Syndrome Occurrence. *International Journal of Molecular Sciences*.

[33] Chan C, Puckridge P, Ullah S, Delaney C, Spark JI (2014). Neutrophil-lymphocyte ratio as a prognostic marker of outcome in infrapopliteal percutaneous interventions for critical limb ischemia. *Journal of Vascular Surgery*.

[34] Kalay N, Dogdu O, Koc F, Yarlioglues M, Ardic I, Akpek M (2012). Hematologic parameters and angiographic progression of coronary atherosclerosis. *Angiology*.

[35] Taşoğlu I, Turak O, Nazli Y, Ozcan F, Colak N, Sahin S (2014). Preoperative neutrophil-lymphocyte ratio and saphenous vein graft patency after coronary artery bypass grafting. *Clinical and Applied Thrombosis/hemostasis: Official Journal of the International Academy of Clinical and Applied Thrombosis/Hemostasis*.

[36] Gibson PH, Croal BL, Cuthbertson BH, Small GR, Ifezulike AI, Gibson G (2007). Preoperative neutrophil-lymphocyte ratio and outcome from coronary artery bypass grafting. *American Heart Journal*.

[37] Erdoğan M, Erdöl MA, Öztürk S, Durmaz T (2020). Systemic immune-inflammation index is a novel marker to predict functionally significant coronary artery stenosis. *Biomarkers in Medicine*.

[38] Liu Y, Ye T, Chen L, Jin T, Sheng Y, Wu G (2021). Systemic immune-inflammation index predicts the severity of coronary stenosis in patients with coronary heart disease. *Coronary Artery Disease*.

[39] Yang YL, Wu CH, Hsu PF, Chen SC, Huang SS, Chan WL (2020). Systemic immune-inflammation index (SII) predicted clinical outcome in patients with coronary artery disease. *European Journal of Clinical Investigation*.

[40] Han K, Shi D, Yang L, Wang Z, Li Y, Gao F (2022). Prognostic value of systemic inflammatory response index in patients with acute coronary syndrome undergoing percutaneous coronary intervention. *Annals of Medicine*.

[41] Xiu WJ, Zheng YY, Wu TT, Hou XG, Yang Y, Ma YT (2022). Hemoglobin-to-Red-Cell Distribution Width Ratio Is a Novel Predictor of Long-Term Patient Outcomes After Percutaneous Coronary Intervention: A Retrospective Cohort Study. *Frontiers in Cardiovascular Medicine*.

[42] Kordzadeh A, Malietzis G, Browne T, Prionidis I, Panayiotopoulos YP (2015). Neutrophil to lymphocyte ratio (NLR) of five predicts 30-day morbidity in ruptured abdominal aortic aneurysms (rAAA): a retrospective cohort study. *International Journal of Surgery (London, England)*.

[43] Sakalihasan N, Limet R, Defawe OD (2005). Abdominal aortic aneurysm. *Lancet (London, England)*.

[44] Shimizu K, Libby P, Mitchell RN (2005). Local cytokine environments drive aneurysm formation in allografted aortas. *Trends in Cardiovascular Medicine*.

[45] Piacentini L, Werba JP, Bono E, Saccu C, Tremoli E, Spirito R (2019). Genome-Wide Expression Profiling Unveils Autoimmune Response Signatures in the Perivascular Adipose Tissue of Abdominal Aortic Aneurysm. *Arteriosclerosis, Thrombosis, and Vascular Biology*.

[46] Cameron SJ, Russell HM, Owens AP (2018). Antithrombotic therapy in abdominal aortic aneurysm: beneficial or detrimental. *Blood*.

[47] Soehnlein O (2012). Multiple roles for neutrophils in atherosclerosis. *Circulation Research*.

[48] Fernández-Ruiz I (2019). Neutrophil-driven SMC death destabilizes atherosclerotic plaques. *Nature Reviews. Cardiology*.

[49] Sheridan FM, Cole PG, Ramage D (1996). Leukocyte adhesion to the coronary microvasculature during ischemia and reperfusion in an in vivo canine model. *Circulation*.

[50] Gratchev A, Sobenin I, Orekhov A, Kzhyshkowska J (2012). Monocytes as a diagnostic marker of cardiovascular diseases. *Immunobiology*.

[51] Rogacev KS, Seiler S, Zawada AM, Reichart B, Herath E, Roth D (2011). CD14++CD16+ monocytes and cardiovascular outcome in patients with chronic kidney disease. *European Heart Journal*.

[52] Appleton ND, Bailey DM, Morris-Stiff G, Lewis MH (2014). Neutrophil to lymphocyte ratio predicts perioperative mortality following open elective repair of abdominal aortic aneurysms. *Vascular and Endovascular Surgery*.

[53] Gong S, Gao X, Xu F, Shang Z, Li S, Chen W (2018). Association of lymphocyte to monocyte ratio with severity of coronary artery disease. *Medicine*.

[54] Wang RH, Wen WX, Jiang ZP, Du ZP, Ma ZH, Lu AL (2023). The clinical value of neutrophil-to-lymphocyte ratio (NLR), systemic immune-inflammation index (SII), platelet-to-lymphocyte ratio (PLR) and systemic inflammation response index (SIRI) for predicting the occurrence and severity of pneumonia in patients with intracerebral hemorrhage. *Frontiers in Immunology*.

[55] Bozza MT, Jeney V (2020). Pro-inflammatory Actions of Heme and Other Hemoglobin-Derived DAMPs. *Frontiers in Immunology*.

[56] Dakour-Aridi H, Nejim B, Locham S, Alshwaily W, Malas MB (2019). Anemia and postoperative outcomes after open and endovascular repair of intact abdominal aortic aneurysms. *Journal of Vascular Surgery*.

[57] Diehm N, Benenati JF, Becker GJ, Quesada R, Tsoukas AI, Katzen BT (2007). Anemia is associated with abdominal aortic aneurysm (AAA) size and decreased long-term survival after endovascular AAA repair. *Journal of Vascular Surgery*.

[58] Ridker PM, Rifai N, Clearfield M, Downs JR, Weis SE, Miles JS (2001). Measurement of C-reactive protein for the targeting of statin therapy in the primary prevention of acute coronary events. *The New England Journal of Medicine*.

[59] Sesso HD, Buring JE, Rifai N, Blake GJ, Gaziano JM, Ridker PM (2003). C-reactive protein and the risk of developing hypertension. *JAMA*.

[60] Förhécz Z, Gombos T, Borgulya G, Pozsonyi Z, Prohászka Z, Jánoskuti L (2009). Red cell distribution width in heart failure: prediction of clinical events and relationship with markers of ineffective erythropoiesis, inflammation, renal function, and nutritional state. *American Heart Journal*.

[61] Vayá A, Sarnago A, Fuster O, Alis R, Romagnoli M (2015). Influence of inflammatory and lipidic parameters on red blood cell distribution width in a healthy population. *Clinical Hemorheology and Microcirculation*.

[62] Dinkla S, van Eijk LT, Fuchs B, Schiller J, Joosten I, Brock R (2016). Inflammation-associated changes in lipid composition and the organization of the erythrocyte membrane. *BBA Clinical*.

[63] Lange A, Kostadinova L, Damjanovska S, Gad I, Syed S, Siddiqui H (2023). Red Cell Distribution Width and Absolute Lymphocyte Count Associate With Biomarkers of Inflammation and Subsequent Mortality in Rheumatoid Arthritis. *The Journal of Rheumatology*.

[64] Hou H, Sun T, Li C, Li Y, Guo Z, Wang W (2017). An overall and dose-response meta-analysis of red blood cell distribution width and CVD outcomes. *Scientific Reports*.

[65] Qu J, Zhou T, Xue M, Sun H, Shen Y, Chen Y (2021). Correlation Analysis of Hemoglobin-to-Red Blood Cell Distribution Width Ratio and Frailty in Elderly Patients With Coronary Heart Disease. *Frontiers in Cardiovascular Medicine*.

[66] Chen X, Wang S, Yang J, Wang X, Yang L, Zhou J (2023). The predictive value of hematological inflammatory markers for acute kidney injury and mortality in adults with hemophagocytic Lymphohistiocytosis: A retrospective analysis of 585 patients. *International Immunopharmacology*.

[67] Nishijima TF, Deal AM, Williams GR, Guerard EJ, Nyrop KA, Muss HB (2017). Frailty and inflammatory markers in older adults with cancer. *Aging*.

[68] Su YC, Wen SC, Li CC, Su HC, Ke HL, Li WM (2021). Low Hemoglobin-to-Red Cell Distribution Width Ratio Is Associated with Disease Progression and Poor Prognosis in Upper Tract Urothelial Carcinoma. *Biomedicines*.

